# HLC Pair Suppression as a Risk Factor for Bacterial Bloodstream Infections and Early Mortality in Newly Diagnosed Intact Immunoglobulin Multiple Myeloma Patients

**DOI:** 10.3389/fonc.2021.599532

**Published:** 2021-03-09

**Authors:** Jose Luis Garcia de Veas Silva, Maria Trinidad Gonzalez Cejudo, Alberto Garcia Perojil Jimenez, Maria del Señor Garcia Lopez Velez, Rafael Garcia Rios Tamayo, Carmen Garcia Bermudo Guitarte, Tomas Garcia De Haro Muñoz

**Affiliations:** ^1^ Department of Laboratory Medicine, Hospital Universitario San Cecilio, Granada, Spain; ^2^ Department of Hematology, Hospital Universitario Virgen de las Nieves, Granada, Spain; ^3^ Department of Clinical Biochemistry, Hospital Universitario Virgen Macarena, Seville, Spain

**Keywords:** myeloma, prognostic factor, HLC (heavy/light chain), infection, immunoparesis

## Abstract

Despite the outstanding progresses in Multiple Myeloma treatment options in the last decades, it remains an incurable disease nowadays. Infectious events are a complication due to an impaired immune system associated with MM, sometimes a life-threatening one, particularly on the first months after the diagnosis. Both the underlying disease and treatment can contribute to the infection risk, so a biomarker that assess this risk could be highly relevant for a more tailored management of the patient. The measurement of the heavy+light chain (HLC) pairs of immunoglobulins in serum allows the quantification of both the monoclonal component and the non-monoclonal immunoglobulin of the same isotype. This approach has demonstrated high sensitivity for the detection of the clonality and prognostic value for MM. HLC pair suppression itself has prognostic power and it has been proposed to be a reflection of the immune system’ attempt to control the tumor. In this study we evaluated the impact of the HLC pair suppression on the rate of bloodstream infections (BSI) and early death in 115 newly diagnosed MM patients. Twenty-one percent of the patients suffered a BSI in the first 6 months after diagnosis, of which 58% died within this period, accounting to 67% of the early deaths in global and highlighting the major impact of infections on MM patients in a “real world” setting. Severe HLC pair suppression identified patients with a higher risk of early BSI (HR: 6,97, p=0,009), and extreme HLC pair suppression together with BSI event and age >65 were independent risk factors for early death (p<0,001). Based on these factors, a stratification model was generated to allow identify patients at a higher risk of early death and poorer OS, with an apparently better performance than the ISS on the early death context. In conclusion, HLC pair suppression associates with both a higher risk of life-threatening early infection and early death in newly diagnosed MM patients. Patients older than 65 with extreme HLC pair suppression and BSI are at a high risk of early death, and thus patients presenting with these criteria have a very adverse prognosis.

## Introduction

Infection is an important cause of morbidity and mortality in severely immunocompromised patients with haematological malignancies undergoing chemotherapy or stem cell transplantation such as multiple myeloma (MM) (1). MM is characterized by a B-cell dysfunction where the patient´s immunoparesis status predisposes to the development of infections (2, 3). Infections represent a major challenge for clinicians caring for MM patients, especially in elderly patients that are highly susceptible to infections (4) and are characterized by presenting complications that increases the risk of mortality (5–7). The primary defect is B cell immunodeficiency, manifested by hypogammaglobulinemia and an increased risk of infection caused by encapsulated bacteria, including Streptococcus pneumoniae and Haemophilus influenzae (8). MM patients have a 7-fold increased risk of infection compared to patients without a malignancy (BLIMARK 2015) and an 11-fold increased risk during the first year following diagnosis (9). In the prevention of infections, clinicians should consider the tumour and host related factors with particular emphasis on disease and age-related organ dysfunction (10). Furthermore, newly diagnosed MM patients are at high risk of very early mortality during the first 2 months after diagnosis when therapy-related adverse effects occur before a significate reduction of tumour load is achieved. About 45% of early mortality after diagnosis is attributed to infections. Respiratory and urinary tracts are the most frequent sites of infection with approximately half of the events associated with hospitalization. Nevertheless, early mortality cannot be accurately predicted by presenting prognostic features (11). During the first 6 months after diagnosis, the risk of infection ranges from 20% to 55% and 10% to 25% of the deaths occurs during this period (12–16). In this context, it would be useful to have access to biomarkers that could accurately predict the risk of infection and early mortality associated to this cause. The quantification of heavy+light chain (HLC) pairs of immunoglobulins in serum by the Hevylite^®^ immunoassay makes possible the precise measurement of monoclonal and non-monoclonal immunoglobulins of the same isotype but of different light chains (i.e. IgG-Kappa and IgG-Lambda in a patient with IgG-Kappa MM). In addition to the more accurately quantification of the M-protein compared to serum protein electrophoresis (17), the assay informs on the suppression of the same isotype non-monoclonal immunoglobulin heavy+light chains pair (i.e. suppression of the IgG-Lambda in an IgG-Kappa MM patient). This type of immunosuppression, which we termed HLC pair suppression, has been independently associated with poor prognosis in MM (18–20). To our knowledge, the impact of HLC pair suppression as a risk factor for bloodstream infections has not been previously evaluated using a cohort of MM patients. The objective of this study is to evaluate HLC pair suppression as a risk factor for bloodstream infections and early death in MM patients and compare it with the classical immunoparesis, i.e., suppression of the immunoglobulins not related to the monoclonal isotype (i.e. reduction of IgA and/or IgM in an IgG MM patient).

## Patients and Methods

### Patients

A total of 115 consecutive newly diagnosed intact immunoglobulin MM patients with measurable disease attending our center from 2013 to 2017 were retrospectively included in the study. Patients with oligosecretory disease or classified as Bence Jones MM were excluded. The patients were diagnosed and followed by experienced Hematologists in monoclonal gammopathies using the IMWG criteria (21). This study is part of the project “HEVYLITE” approved with the codes 0697-N-16 and 0697-M1-17 by the Local Ethics Committee. Permission was obtained from the Department of Clinical Documentation to collect data from the medical records. All the data of the study were collected and analysed anonymously.

### Clinical Data of the Patients

Clinical data of the patients including sex, age, myeloma subtype, disease stage based on ISS, haemoglobin, serum beta-2 microglobulin (B2M), serum calcium, serum creatinine, serum albumin, serum immunoglobulin, immunoparesis of non-monoclonal immunoglobulins, serum free light chains (sFLC), immunoglobulin heavy+light chain (HLC) pairs, serum lactate dehydrogenase (LDH), presence of lytic bone lesions, bone marrow (BM) plasma cell infiltration, serum protein electrophoresis (SPE), M-protein concentration and immunofixation results were obtained at the time of diagnosis. FISH cytogenetics studies included evaluation of del 13q, del 17p, t(11;14), t(4;14) and t(14;16). Quantitative variables were categorized as follow: age >65 years, highly abnormal HLC ratio <0.022 or >45 (22), Highly abnormal sFLC ratio <0.03 or >32 (23), haemoglobin <10 g/dl, calcium >11 mg/dl, creatinine >2 mg/dl, B2M >5.5 mg/L, albumin <3.5 g/dl, BM plasma cell infiltration>20% and LDH>250 U/L. Haemoglobin was measured on a Sysmex 2000 analyzer; serum immunoglobulins, albumin, LDH, B2M, calcium, creatinine were measured on a COBAS 6000 analyzer. Serum free light chains were measured by turbidimetry using Freelite™ (The Binding Site Group Ltd, Birmingham, UK) on a SPA PLUS Specialist Protein Analyzer.

### Immunoglobulin Heavy+Light Chain Pairs and Immunoparesis Definitions

Immunoglobulin heavy+light chain pairs (IgG-Kappa/IgG-Lambda and IgA-Kappa/IgA-Lambda) were measured on a SPAPLUS Specialist Protein Analyser by turbidimetry using the Hevylite^®^ immunoassay (The Binding Site Group Ltd, Birmingham, UK) based on polyclonal antibodies directed against a unique junctional epitope between heavy chain and light chain constant regions of intact immunoglobulins. The HLC reference ranges used were those provided by the supplier: IgG-Kappa=3.84–12.07 g/L; IgG-Lambda=1.91–6.74 g/L; IgG-Kappa/IgG-Lambda=1.12–3.21; IgA-Kappa=0.57–2.08 g/L; IgA-Lambda=0.44–2.04 g/L; IgA-Kappa/IgA-Lambda=0.78–1.94. Based on the results by Ludwig et al. (18), severe HLC-matched pair suppression was defined as a >50% reduction below the lower limit of normal (LLN) ranges of the respective immunoglobulins (i.e. IgG-Kappa <1.92 g/L, IgG-Lambda <0.95 g/L, IgA-Kappa <0.28 g/L, IgA-Lambda <0.22 g/L). Systemic immunoparesis (SI) was defined as either one or two non-involved polyclonal isotypes (i.e. IgA and/or IgM in an IgG MM patient) being >50% below the LLN of the respective isotypes (IgG <350 mg/dl, IgA <35 mg/dl, IgM <20 mg/dl). Furthermore, extreme HLC-matched pair suppression was defined when a >95% reduction below the LLN of respective isotypes was observed (i.e. IgG-Kappa <0.192 g/dl, IgG-Lambda <0.095 g/dl, IgA-Kappa <0.028 g/dl, and IgA-Lambda <0.022 g/dl).

### Bloodstream Infection Definition

Bloodstream infection (BSI) was defined as a positive blood culture related to a febrile episode according to the Centres for Disease Control and Prevention/National Healthcare Safety Network (CDC/NHSN) guidelines (24).

### Endpoints

In order to explore the impact of HLC pair suppression and SI on bloodstream infections and early mortality, only events within 6 months (180 days) from diagnosis were documented. Patients were stratified in two groups according to the development of infections: BSI and non-BSI group. The primary endpoint of this study was the early mortality within 6 months (180 days) from diagnosis of the patients. Overall survival (OS) was defined as the time from initial diagnosis to death or last follow-up.

### Statistical Analysis

Pearson’s chi-squared test for categorical variables was used to compare patient’s characteristics among groups. OS was estimated using the method of Kaplan and Meier and the survival curves were compared using the Log-Rank test. Clinical and laboratory variables were evaluated for their impact on patient’s outcome. Cox regression proportional hazards model was used for univariate and multivariate analysis to study the influence of prognostic factors in BSI and early mortality. Multivariate analysis was performed including variables with a *p value <*0.2 obtained in univariate analysis. The Akaike information criterion (AIC) was calculated for every prognostic model. AIC provided a qualitative comparison of models, where a lower AIC indicates better model fit (25). A *p value <*0.05 was considered to be statistically significant for all comparisons. All p values are two-sided and confidence intervals refer to 95% boundaries.

## Results

### Patients Characteristics

The baseline characteristics of the 115 patients included in the study are shown in **Table 1**. Exceptions were FISH cytogenetics data and percentage of plasma cells in bone marrow with data available only for 58 and 106 of the patients, respectively. The median age of the patients was 68 years (range: 56–77 years), 55% were >65 years old, and 48 patients (42%) were female. The MM isotype was IgG in 76 patients (66%) and IgA in 39 patients (34%): 49 IgG-Kappa, 27 IgG-Lambda, 21 IgA-Kappa and 18 IgA-Lambda. All 115 patients presented with an abnormal HLC ratio at diagnosis. According to ISS, 25 patients (22%) were stage 1, 25 (22%) were stage 2 and 65 (56%) were stage 3. The induction regimens were Bortezomib-Dexamethasone with or without Cyclophosphamide (53 patients), Bortezomib-Dexamethasone-Cyclophosphamide followed by Lenalidomide-Dexamethasone (8), Lenalidomide-Dexamethasone (6), Bortezomib-Lenalidomide-Dexamethasone-Cyclophosphamide (16) and Bortezomib-Melphalan-Prednisone (15). Seventeen patients did not received treatment due to early death.

**Table 1:** Baseline characteristic of the 115 multiple myeloma patients stratified by the presence of bloodstream infections.

Parameter	Al patients (N=115)	Patients with non-BSI (n=91, 79%)	Patients with BSI (n=24, 21%)	P value
Age (years)	68 (56-77)	66 (55-76)	73 (58-80)	0.05
Age >65 years	63 (55%)	46 (51%)	17 (71%)	0.07 NS
Gender				
Male	67 (58%)	54 (59%)	13 (54%)	0.6 NS
Female	48 (42%)	37 (41%)	11 (46%)	
Myeloma isotype				0.3 NS
IgG	76 (66%)	59 (65%)	17 (71%)	
IgA	39 (34%)	32 (35%)	7 (29%)	
IgG (IgG-K/IgG-L)	76 (49/27)	59 (41/18)	17 (8/9)	
IgA (IgA-k/IgA-L)	39 (21/18)	32 (17/15)	7 (4/3)	
Severe HLC pair suppression	73 (64%)	51 (56%)	22 (92%)	0.001
Extreme HLC pair suppression	21 (18%)	11 (12%)	10 (42%)	0.001
Severe systemic immunoparesis	53 (46%)	39 (43%)	14 (58%)	0.1 NS
ISS stage I	25 (22%)	22 (24%)	3 (13%)	0.1 NS
ISS stage II	25 (22%)	22 (24%)	3 (13%)	
ISS stage III	65 (56%)	47 (52%)	18 (74%)	
Bone lesions (%)	85 (74)	65 (71%)	20 (83%)	0.1 NS
FISH Cytogenetics*	13 (22%)	9 (18%)	4 (57%)	0.019
HLC ratio <0.022 or >45	67 (58%)	48 (53%)	19 (79%)	0.02
FLC ratio <0.03 or >32	77 (67%)	61 (67%)	16 (67%)	0.9 NS
B2M >5.5 mg/L	63 (54%)	45 (50%)	18 (75%)	0.03
Albumin <3.5 g/dl	61 (53%)	43 (47%)	18 (75%)	0.015
Haemoglobin <10 g/dl	49 (43%)	36 (39%)	13 (54%)	0.2 NS
Calcium >11 mg/dl	20 (17%)	16 (18%)	4 (17%)	0.9 NS
Creatinine >2 mg/dl	30 (26%)	19 (21%)	11 (46%)	0.013
LDH >250 U/L	26 (23%)	15 (16%)	11 (46%)	0.002
BMPC >20%**	52 (49%)	43 (51%)	9 (43%)	0.5 NS
Early death(%)	21 (18%)	7 (8%)	14 (58%)	<0.0001

Qualitative data expressed as n(%). Quantitative data expressed as median (interquartile range).

Median follow-up of the patients was 15 months (range: 6.5–27.4).

* Data only available for 58 patients. FISH Cytogenetics data includes analysis of del 13q, del 17p and R(IgH).

** Data only available for 106 patients. NS, Not significant.

Twenty-four patients (21%) developed BSI within 6 months of diagnosis: Within patients presenting BSI group, pathogens were isolated in blood culture: 9 Gram-negative bacilli (37%) and 15 Gram-positive bacteria (63%). No fungi were isolated during the period of study and none of the patients presented mixed pathogens in blood culture. The most common frequently found pathogen was Streptococcus pneumoniae (n=8) followed by Klebsiella pneumoniae (n=5), Escherichia coli (n=4), Staphylococcus aureus (n=4), Staphylococcus coagulase negative (n=2), and Enterococcus faecium (n=1). Comparing the BSI group with the non-BSI group, in the first group we observe a greater incidence of HLC pair suppression (both severe and extreme) (p=0.001) and highly abnormal HLC ratios (p=0.02). BSI group also showed a higher percentage of patients with cytogenetic alterations identified by FISH (p=0.019), and with highly altered B2M (p=0.03) and albumin<3.5 g/dl (p=0.015), altered renal function (creatinine >2 mg/dl, p=0.013) and higher levels of LDH (p=0.002). No significant differences were observed between groups in regard to age, gender, immunoglobulin monoclonal isotype, ISS stage, SI, highly abnormal sFLC ratio, haemoglobin, calcium, BM plasma cell infiltration, and presence of bone lesions.

### Risk Factors for the Development of BSI in Multiple Myeloma Patients

Univariate analysis showed age, severe and extreme HLC-pair suppression, FISH cytogenetics, highly altered HLC ratio, elevated B2M, decreased albumin, altered renal function (creatinine >2 mg/dl) and an altered LDH as possible risk factors associated with the development of BSI in the patients. No association was found with other analysed parameters (**Table 2**). On a multivariate analysis is performed, only severe HLC-pair suppression (HR=8.12, p=0.005), increased creatinine (HR=2.61, p=0.02), and elevated LDH (HR=2.98, p=0.01) come out as independent risk factors for BSI (**Table 2**).

**Table 2:** Univariate and multivariate analysis for risk factors associated with the development of bloodstream infections.

Parameter	Univariate analysis		Multivariate analysis	
	HR (95% CI)	P value	HR (95% CI)	P value
Age >65 years	2.50 (1.04–6.03)	**0.042**	2.15 (0.87-5.30)	0.09
Gender (female vs. male)	1.20 (0.54–2.67)	0.6 NS	–	–
Myeloma isotype (IgG vs. IgA)	1.19 (0.49–2.87)	0.7 NS	–	–
Severe HLC-matched pair suppression	7.19 (1.69–30.57)	**0.002**	6.97 (1,62-29,94)	**0.009**
Extreme HLC-matched pair suppression	4.85 (2,13–11,09)	**0.001**		
Severe systemic immunoparesis	1.71 (0.76–3.85)	0.1 NS	–	–
ISS stage II vs. I	1.08 (0.27–5.33)	0.9 NS	–	–
ISS stage III vs. I	2.69 (0.79–9.14)	0.1 NS	–	–
FISH Citogenetics*	5.56 (1.24–24.91)	**0.025**	–	–
HLC ratio <0.022 or >45	3.35 (1.25–8.98)	**0.016**	–	–
FLC ratio <0.03 or >32	0.94 (0.40–2.20)	0.9 NS	–	–
B2M >5.5 mg/L	2.82 (1.12–7.11)	**0.028**	–	–
Albumin <3.5 g/dl	3.42 (1.35–8.63)	**0.01**	–	–
Hemogloblin <10 g/dl	1.85 (0.83–4.14)	0.1 NS	–	–
Calcium >10.5 mg/dl	1.07 (0.37–3.14)	0.9 NS	–	–
Creatinine >2 mg/dl	2.68 (1.20–5.99)	**0.016**	2.30 (1.01–5.25)	**0.04**
LDH >250 U/L	3.41 (1.53–7.63)	**0.003**	3.34 (1.47–7.59)	**0.04**
BMPC >20%**	0.74 (0.31–1.76)	0.5 NS	–	–

* Data only available for 58 patients. FISH Cytogenetics data includes analysis of del 13q, del 17p and R(IgH).

** Data only available for 106 patients. NS, Not significant. The bold values in the tables are the “significant values” in thestatistical analysis.

**Figure 1:**
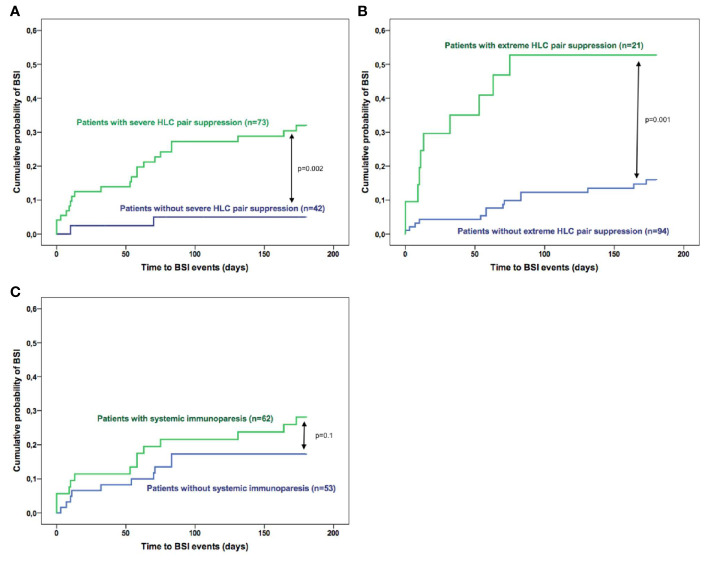
Risk of bloodstream infections in multiple myeloma according to the immunoparesis status of the patients based on **(A)** severe HLC pair suppression; **(B)** extreme HLC pair suppression and **(C)** systemic immunoparesis.

Regarding the immunoparesis status of the patients, the risk of BSI was significantly higher among patients with severe HLC pair suppression *versus* those without suppression (32% vs. 5% at 6 months, respectively; HR: 7.19, p=0.002, **Figure 1A**). Similar results were found when the patients were stratified according to extreme HLC pair suppression with 53% of the patients with extreme HLC pair suppression developing BSI within 6 months of diagnosis and 16% of the patients with and without extreme HLC pair suppression (HR: 4.85, p=0.001, **Figure 1B**). By contrast, no significant association was observed between severe SI and risk of infection (p=0.1, **Figure 1C**).

### Outcome of the Patients and Factors Associated With Early Mortality

The median follow-up of the 115 patients was 15 months (range: 6.5–27.4). Twenty-one patients out of 115 (18%) died within 6 months of diagnosis (**Table 1**). Infection was the leading cause of death accounting for 67% of cases (14 patients). The rate of death was superior on the BSI group when compared to the non-BSI group (58% vs. 8%, respectively, p<0.0001, **Table 1**). Univariate analysis showed that several parameters were significantly associated with OS within 6 months after diagnosis (**Table 3**) but multivariate analysis identified only age (HR: 3.71, p=0.03), extreme HLC-matched pair suppression (HR: 5.14, p=0.001) and BSI (HR: 5.12, p=0.001) as being factors that independently predict early mortality.

**Table 3:** Univariate and multivariate analysis for risk factors associated with early mortality.

Parameter	Univariate analysis		Multivariate analysis	
	HR (95% CI)	P value	HR (95% CI)	P value
Age >65 years	6.09 (1.79–20.68)	**0.004**	3.71 (1.05–13.08)	**0.03**
Gender (female vs. male)	1.03 (0.43–2.43)	0.9 NS	–	–
Myeloma isotype (IgG vs. IgA)	0.64 (0.27–1.52)	0.3 NS	–	–
Severe HLC-matched pair suppression	3,65 (1,07–12,38)	**0.026**	–	–
Extreme HLC-matched pair suppression	10.00 (4,11–24,32)	**<0.001**	5.14 (1.92–13.76)	**0.001**
Severe systemic immunoparesis	1.51 (0.63–3.57)	0.4 NS	–	–
BSI	9.57 (3.84–23.83)	**<0.001**	5.12 (1.89–13.87)	**0.001**
ISS stage II vs. I	2.02 (0.18–22.26)	0.6 NS	–	–
ISS stage III vs. I	7.56 (1.01–56.62)	**0.049**	–	–
FISH Citogenetics*	4.06 (1.17–14.06)	**0.03**	–	–
HLC ratio <0.022 or >45	7.78 (1.81–33.43)	**0.006**	–	–
FLC ratio <0.03 or >32	0.99 (0.39–2.44)	0.9 NS	–	–
B2M >5.5 mg/L	5.42 (1.59–18.42)	**0.007**	–	–
Albumin <3.5 g/dl	9.81 (2.28–42.12)	**0.002**	–	–
Hemogloblin <10 g/dl	2.43 (1.01–5.87)	**0.04**	–	–
Calcium >10.5 mg/dl	1.56 (0.57–4.27)	0.4 NS	–	–
Creatinine >2 mg/dl	2.75 (1.17–6.47)	**0.021**		
LDH >250 U/L	1.87 (0.76–4.64)	0.2 NS		
BMPC >20%**	0.54 (0.20–1.46)	0.2 NS	–	–

* Data only available for 58 patients. FISH Cytogenetics data includes analysis of del 13q, del 17p and R(IgH).

** Data only available for 106 patients. NS, Not significant. The bold values in the tables are the “significant values” in thestatistical analysis.

**Figure 2:**
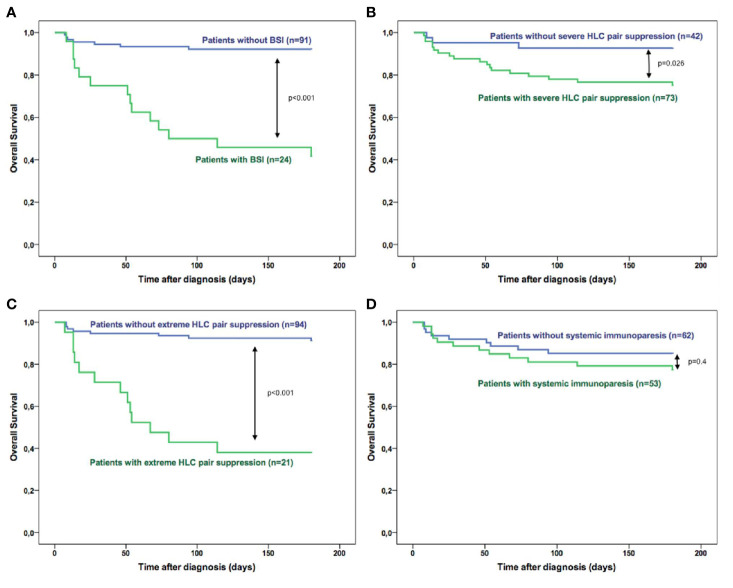
Overall survival of multiple myeloma patients during the first 6 months after diagnosis according to **(A)** the presence of bloodstream infections; **(B)** severe HLC pair suppression; **(C)** extreme HLC pair suppression and **(D)** systemic immunoparesis in the patients at diagnosis.

Survival analysis showed that patients with BSI had worse survival outcome than those without BSI: OS was 42% and 92%, respectively (HR: 9.57, p<0.001, **Figure 2A**). Also, patients with HLC pair suppression had shorter OS compared to those without (75% vs. 93% and 38% vs. 91% for severe (p=0.026, **Figure 2B**) and extreme (p<0.001, **Figure 2C**) HLC pair suppression, respectively). Conversely, no significant association was found between SI and early mortality (p=0.4, **Figure 2D**).

### Prognostics Models for Early Mortality

**Figure 3:**
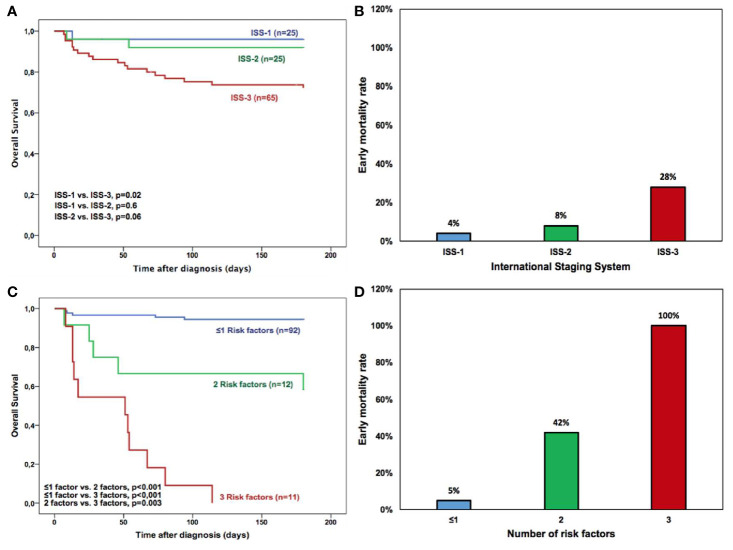
Overall survival of all multiple myeloma patients based on **(A)** International Staging System (ISS) and **(C)** New stratification risk model where age >65 years, extreme HLC-matched pair suppression and bloodstream infection (BSI) were defined as adverse risk factors. Overall Survival was significantly different among patients with ≤1, 2, or 3 adverse risk factors. Early mortality rate of multiple myeloma patients according to **(B)** International Staging System and **(D)** New stratification risk model based on ≤1, 2, or 3 adverse risk factors. Age >65 years, extreme HLC-matched pair suppression and bloodstream infection (BSI) were defined as adverse risk factors.

ISS model did not present prognostic value for early mortality in our patient cohort (**Table 3** and **Figure 3A**). Considering the parameters of adverse prognostic risk factors identified by multivariate analysis (age >65 years, extreme HLC-matched pair suppression and bloodstream infections) a risk model was generated based on the presence of either ≤1, 2, or 3 adverse risk factors (**Figure 3C**). The accumulation of risk factors identified groups of patients with increasing risk of early mortality at 6 months: 5%, 42%, and 100% risk of early mortality at 6 months (p<0.001, **Figure 3C**). The OS was significantly shortened as the number of risk factors increased (**Figure 3D**). The prognostic power of both models for identifying patients at high risk of early mortality was compared using the AIC values. The model based on adverse risk factors had a lower AIC (AIC=155.4) than the ISS model (AIC=188.2) resulting in a better model fit than ISS.

## Discussion

Although the long-term survival of patients with haematological malignancies has improved during the last few decades, it has been recognised that infections are one of the most serious threats to these patients and where bacteraemia is frequent and is associated with high mortality rates (25–28). In MM patients, bacteria are the most frequent aetiology agents although invasive fungal infections caused by molds have been increasingly reported (10). Our data is in agreement with previous studies indicating that gram-positive bacteria account for more than 50 percent of such infections (29, 30) with S. pneumoniae and Staphylococcus aureus being the most common pathogens. Among gram-negative bacteria, Escherichia coli is the leading pathogen. Anticipating the risk of infection allows the clinicians to act accordingly. Thus, it is essential to identify the factors associated to infection and early death so that adjusted preventive strategies can be developed/implemented. In the current study, during the six first months after diagnosis, BSI occurred in 21% of all MM patients and the rate of early mortality was 18% with 67% being attributable to infections, therefore depicting a major cause of death in this population. The results obtained here are in line with previous studies (12–15). An UK study, including 3107 MM patients reported a 10% death rate within 60 days following diagnosis (11), being infection the most frequent cause (45%) and with pneumonia accounting for 66% of bacterial infections. More recently a study conducted in the United States by Costa et al., in 2015 reported 28.6% mortality within the first year following diagnosis (31). Also, we previously reported a 10,6% and 20% of early mortality at 2 and 6 months after diagnosis, respectively, on a large population-based cohort of newly diagnosed MM patients from our Hospital. Respiratory disease associated with very early mortality at the first 2 months after diagnosis (16). MM patients are prone to infection because of immune dysfunction caused either by the disease, the effects of chemotherapy or advanced age (10). Hargreaves et al. found that the immunosuppression of non-paraprotein IgG or IgA was significantly associated with at least one episode of serious infection in MM patients at plateau phase (3). Suppression of non-paraprotein immunoglobulins was also described as in independent risk factor for progression in patients with asymptomatic monoclonal gammopathies (32). This immunosuppressed status of the patients has been traditionally defined as systemic immunoparesis based on measurement of serum immunoglobulins levels: IgG, IgA, and IgM. Nowadays, the HLC assay can distinguish between the involved and uninvolved immunoglobulins of a same isotype but of alternative light chains (17). For instance, in an IgA-Kappa MM patient, this assay allows a more precise measurement of the monoclonal (IgA-Kappa) and non-monoclonal (IgA-Lambda) immunoglobulins, separately. To the best of our knowledge, this is the first study were the immunoparesis status of the patient/suppression of non-clonal immunoglobulin/clonal immunoparesis at diagnosis has been evaluated as risk factor for BSI and early mortality in MM patients. The results here presented showed that the suppression of non-clonal same isotype immunoglobulin/severe HLC-matched pair suppression, which we defined as clonal immunoparesis is a promising factor for determining risk of infection in MM patients. Patients with severe HLC-matched pair suppression presented a greater risk of BSI compared to those without suppression (HR: 6.97, p=0.009). On the other hand, the classical immunoparesis termed systemic immunoparesis, defined by the suppression of one or two of the non-involved immunoglobulins did not associate significantly with risk of infection. These findings show that the clonal immunoparesis could better represent a greater degree of immunosuppression than the systemic immunoparesis. As once hypothesized by Ludwig et al. (18), the HLC-matched pair suppression likely reflects the immune system´s attempt to confine the myeloma clones affecting both involved clonal and non-involved polyclonal plasma cells of the same isotype (18).

Additionally, elevated levels of LDH and creatinine were the only other independent risk factors for development of infections. These features are associated with disease severity. Elevated serum levels of LDH is indicative of an aggressive disease suggesting a high tumour burden and the presence of occult extraosseous disease with high levels associated with shorter OS (33, 34). Furthermore, high levels of creatinine (>2 mg/dl) characteristic of renal dysfunction in the MM patients has been considered a risk factor for infection (10). A recent analysis of the FIRST clinical trial identified a set of risk factors associated with early infections related to the MM treatment which include LDH and creatinine, among others, and more interestingly, in an exploratory analysis they found that patients with baseline immunoparesis had increased risk of grade >3 early infections (35).

Interestingly, in the present study, extremely HLC-matched pair suppression, age above 65 years old, and the presence of BSI were identified as risk factors for early mortality, while serum levels of albumin, B2M, and LDH were not. Although albumin and B2M are the basis for ISS, the prognostic impact of this score is associated with OS (27, 36), and not with early mortality. On the other hand, HLC-matched pair suppression and BSI were not studied/considered before as clinical variables of poor prognosis. Although MM remains as an incurable disease, the life expectancy of the patients has improved significantly during the last 2 decades due to the treatment with novel drugs followed by autologous stem cell transplantation (37, 38). Nonetheless, the impact of clinical variables associated with early mortality in the patients is less well known and more studies are needed to identify predicting factors for early mortality instead of overall survival.

The quantification of HLC pairs have been previously shown to have prognostic value in MM patient as well as monitoring patients, especially those with monoclonal proteins difficult to detect by electrophoresis (22, 39, 40), but not in predicting early mortality. Additionally, severe HLC-matched pair suppression has been associated with poor prognosis in previously untreated patients with myeloma and those with relapsed disease with the persistence of HLC-matched pair suppression, being correlated with shorter survival after best response (18, 20). In the current study, the interesting findings about the role of HLC-matched pair suppression on early mortality are novel and have not been described before in the literature.

In this context, a good prognostic stratification system should divide patients in groups with different risk of early mortality. The International Staging System (ISS), based on serum levels of B2M and albumin, is an easy and quickly prognostic model to estimate the probability of survival of newly diagnosed MM patients (41). The legitimacy of this system has sometimes been questioned with the introduction of novel treatment agents (42–44). In the present study, the ISS model did not result as an independent predictor for early mortality in the patients studied. Only the ISS stage 3 had significant prognostic value for survival in univariate analysis (**Table 3**). We found that a risk stratification model that considers age >65 years, extreme HLC suppression and BSI appears more indicative of early mortality when compared to the ISS model. The reason for these discrepancies might be due to the fact that the ISS is a prognostic score based on tumor burden and its importance lies in its ability to discriminate differences in long-term OS. In our previous study, ISS is not relevant until the OS cutoff at 12 months (16). However, we have not tested our model in a long-term follow-up scenario, where the ISS model may come out with a better prognostic capacity. With these findings, the first months after diagnosis should be considered a period of high risk of mortality by the haematologist that have to remain vigilant.

The results suggest that the identification of patients with clonal immunoparesis could help the haematologists to choose the adequate therapeutic and prophylactic strategies aimed to minimize the risk of infection and early death. A strength in our study is that the infection diagnosis is based on laboratory data proving the infectious agents. Furthermore, another important feature of the study is that we included a uniform group of patients that received induction treatment based on novel agents. Among the limitations of the study we can consider the retrospective analysis of the data, the relative small number of patients in the study (n=115), the incomplete data of cytogenetics alterations by FISH and the lack of information about comorbidities.The lack of validation of the risk stratification model obtained could be considered a limitation of the study.

In conclusion, our study showed that the HLC-matched pair suppression is a condition associated with high risk of infections and early mortality in newly diagnosed myeloma patients. Although our data provided important insights in the prognostic of the patients at diagnosis, the validation of these finding with a greater number of patients is necessary to confirm the role of HLC-matched pair suppression as biomarker of bacterial bloodstream infection and early mortality in MM patients. The role HLC-matched pair suppression in viral infections should also be addressed in future studies.

## Data Availability Statement

The datasets presented in this article are not readily available because are part of an extended database where more results and conclusions can be obtained in subsequent studies. Requests to access the datasets should be directed to jose6@outlook.com.

## Ethics Statement

The studies was reviewed and approved by “Ethics Committee of Biomedical of Research of Granada” with the codes of 0697-N-16 0697-M1-17. This study is based in data obtained in routine clinical practice, so written informed concern for participation is not required. All the data of of the study were analyzed anonymously.

## Author Contributions

JV—concept, methodology, statistical analysis, writing. MG—methodology, statistical analysis, writing. AJ—analytical determinations, writing, statistical analysis. ML—methodology, analytical determinations, revision. RT—diagnosis and treatment of patients. CG—manuscript review. TM—manuscript review. All authors contributed to the article and approved the submitted version.

## Conflict of Interest

The authors declare that the research was conducted in the absence of any commercial or financial relationships that could be construed as a potential conflict of interest.
